# Combining genetic and demographic data for prioritizing conservation actions: insights from a threatened fish species

**DOI:** 10.1002/ece3.645

**Published:** 2013-07-09

**Authors:** Ivan Paz-Vinas, Lise Comte, Mathieu Chevalier, Vincent Dubut, Charlotte Veyssiere, Gaël Grenouillet, Geraldine Loot, Simon Blanchet

**Affiliations:** 1 UMR5174 EDB (Laboratoire Évolution & Diversité Biologique), Centre National de la Recherche Scientifique (CNRS), École Nationale de Formation Agronomique (ENFA), Université Paul Sabatier118 route de Narbonne, F-31062, Toulouse Cedex 4, France; 2 UMR 5174 (EDB)UPS, Université de Toulouse118 route de Narbonne, F-31062, Toulouse Cedex, France; 3 Centre National de la Recherche Scientifique (CNRS), Station d'Ecologie Expérimentale du CNRS à MoulisUSR 2936, F-09200, Moulis, France; 4 UMR 5245 EcoLab (Laboratoire Ecologie Fonctionnelle et Environnement), CNRSF-31062, Toulouse, France; 5 IMBE – UMR 7263, Aix-Marseille Université, CNRS, IRDCentre Saint-Charles, Case 36, 3 place Victor Hugo, F-13331, Marseille Cedex 3, France

**Keywords:** Bottleneck, conservation genetics, demographic survey, *Parachondrostoma toxostoma*, rivers, species distribution models, temporal trends

## Abstract

Prioritizing and making efficient conservation plans for threatened populations requires information at both evolutionary and ecological timescales. Nevertheless, few studies integrate multidisciplinary approaches, mainly because of the difficulty for conservationists to assess simultaneously the evolutionary and ecological status of populations. Here, we sought to demonstrate how combining genetic and demographic analyses allows prioritizing and initiating conservation plans. To do so, we combined snapshot microsatellite data and a 30-year-long demographic survey on a threatened freshwater fish species (*Parachondrostoma toxostoma*) at the river basin scale. Our results revealed low levels of genetic diversity and weak effective population sizes (<63 individuals) in all populations. We further detected severe bottlenecks dating back to the last centuries (200–800 years ago), which may explain the differentiation of certain populations. The demographic survey revealed a general decrease in the spatial distribution and abundance of *P. toxostoma* over the last three decades. We conclude that demo-genetic approaches are essential for (1) identifying populations for which both evolutionary and ecological extinction risks are high; and (2) proposing conservation plans targeted toward these at risk populations, and accounting for the evolutionary history of populations. We suggest that demo-genetic approaches should be the norm in conservation practices.

We combined genetic and demographic data from a threatened freshwater fish species (*Parachondrostoma toxostoma*) at the river basin scale for conservation purposes. Genetic diversity and effective population sizes are very low, probably due to the strong genetic bottlenecks detected in this study. The species spatial distribution and abundance also decreased during the last decades.

## Introduction

Prioritizing and making appropriate plans to manage and conserve threatened species is a complex task. Global changes simultaneously affect multiple facets of individual species, making predictions difficult (Margules and Pressey 57; McMahon et al. 59). For instance, global changes such as habitat fragmentation or climate change can affect the genetic diversity (Olivieri et al. 62; Blanchet et al. 7), the demographic dynamics (Julliard et al. 47; Dunham et al. 26), the evolution of life-history traits (Conover et al. 14; Blanchet and Dubut 6), and/or the spatial distribution of species (Parmesan 66; Buisson et al. 10). Accordingly, the conservation biologists’ toolbox includes several methods which emerged from multiple disciplines such as population genetics, population ecology, and biostatistics (Guisan and Zimmermann 41; Green et al. 39; Excoffier and Heckel 28). Nevertheless, most conservation studies focus on a single facet of species health (e.g., the genetic diversity), and hence provide only partial information for biodiversity management and conservation (Frankham 33; Geist 34; Loss et al. 53).

Integrative studies are, however, increasingly acknowledged as being valuable from a conservation standpoint (Purvis and Hector 73; Geist 34; Loss et al. 53). For instance, at the community level, Devictor et al. (22) showed that there was a strong spatial mismatch between phylogenetic, functional, and taxonomic measures of bird biodiversity. These measures provide different but complementary information, suggesting that reserve designs should be optimized accordingly (Devictor et al. 22). Similarly, at the population level, diverse measures classically used to assess the health of a population (e.g., effective population size, abundance, and dispersal rate) provide complementary information that should be integrated into common analyses to set efficient conservation plans (e.g., Osborne et al. 63, 64). For instance, demographic monitoring programs (hereafter, DMPs) provide useful information regarding the ecological status of populations and enable predictions on future distributions under global change scenarios, whereas population genetics studies (hereafter, PGSs) obtain information regarding the evolutionary status of populations and their potential resistance to rapid environmental changes (Smith and Bernatchez 82). Because evolutionary and ecological timescales and processes are sometimes confounded (Carroll et al. 12), it is of prime importance to merge evolutionary and ecological information to (1) identify the populations that need to be prioritized for conservation actions; and (2) implement effective long-term management and conservation of endangered populations (Osborne et al. 64).

The use of population genetics in biodiversity conservation has increased considerably in the last decades (Frankham 33). Low genetic diversity in natural populations has been generally associated with pervasive effects such as inbreeding depression, loss of evolutionary potential, and the accumulation of deleterious mutations (Saccheri et al. 78; Frankham 33). These effects theoretically increase extinction risks, and are expected to be stronger in populations under anthropogenic or natural stresses (Spielman 83). Accordingly, PGSs generally aim at (1) describing the genetic status of populations (i.e., genetic diversity and structure assessed during a snapshot survey, Schwartz et al. 79); (2) identifying historical and contemporary factors affecting the genetic diversity of populations (Manel et al. 56; Dubut et al. 25); and (3) inferring past and contemporary demographic parameters such as effective population sizes (*N*_e_) (Storz and Beaumont 84). Although PGSs provide key information about demographic processes, linking genetics and population demography remains tricky (Osborne et al. 64). For instance, the link between *N*_e_ and census population size (*N*_c_) is notoriously difficult to assess (Luikart et al. 55; Belmar-Lucero et al. 4; Palstra and Fraser 65), and genetic bottlenecks (i.e., strong decreases in *N*_e_) can be detected even in the absence of demographic bottlenecks (Broquet et al. 9; Chikhi et al. 13). Furthermore, the effects of particular threats may be undetected through PGSs due to the lag time that often exists between an ecological cause and its evolutionary consequence (Landguth et al. 50).

Analyses based on demographic data can overcome some of these gaps (Nichols and Williams 61; Lindenmayer et al. 51). DMPs provide information about the current status of populations by allowing the inference of key demographic parameters such as abundance and/or occurrence (Royle and Dorazio 77). Combined with time series analyses, DMPs also permit the investigation of temporal trends and hence the identification of the causes and consequences of population declines or changes in spatial distribution (Daufresne et al. 21). Additionally, these surveys are useful for the early detection of the effects of threats on populations as well as “ecological surprises” (Doak et al. 23), which is notoriously difficult using only PGSs (Julliard et al. 47; Lindenmayer et al. 51). Finally, long-term and large spatial-scale surveys are of prime interest and may allow predictions about the future status of populations in a changing world through the use of species distribution models for instance (Guisan and Zimmermann 41).

In this study, we attempt to demonstrate how combining PGSs and DMPs provides baseline information for prioritizing and initiating management and conservation plans. We focused on an endangered freshwater fish species (i.e., the South-west European nase *Parachondrostoma toxostoma*, Vallot 1837) which is considered vulnerable throughout its restricted native range (i.e., Southern France, Crivelli 19). We used a microsatellite dataset gathered at the river basin scale (i.e., the Garonne river basin, South-Western France) to (1) describe the genetic diversity and structure of *P. toxostoma* populations, and (2) detect and quantify both contemporary and past *N*_e_ (i.e., contraction or reduction in *N*_e_ over time)*,* as well as to date main changes in *N*_e_ following the last glacial maximum (i.e., approximately 10,000 years ago). In parallel, we used a demographic survey performed at the same spatial scale over the last three decades to (3) identify temporal trends in species abundance at the Garonne river basin scale; and (4) assess the current spatial distribution of the species and changes in the distribution over the last three decades.

## Materials and Methods

### Biological model

*Parachondrostoma toxostoma* is a threatened freshwater fish species of the Cyprinidae family endemic to France and Switzerland, where its native range area is restricted to the Rhône, Adour and Garonne river basins. This species is listed as vulnerable in the IUCN red list, in the Annex II of the European Union Habitats Directive and in Appendix III of the Bern Convention (Crivelli 19). The range of the species has been strongly reduced due to water pollution, habitat fragmentation by dams and weirs, artificial water releases and hybridization with a nonnative species, *Chondrostoma nasus* (Costedoat et al. 17). Our study focuses on the Garonne river basin, which hosts the major stock of pure *P. toxostoma* (i.e., not introgressed by the *C. nasus* genome). This highlights the urge for conservation actions directed toward the Garonne drainage in order to preserve the *P. toxostoma* species.

### Population genetics study

#### Sampling design

**Figure 1:**
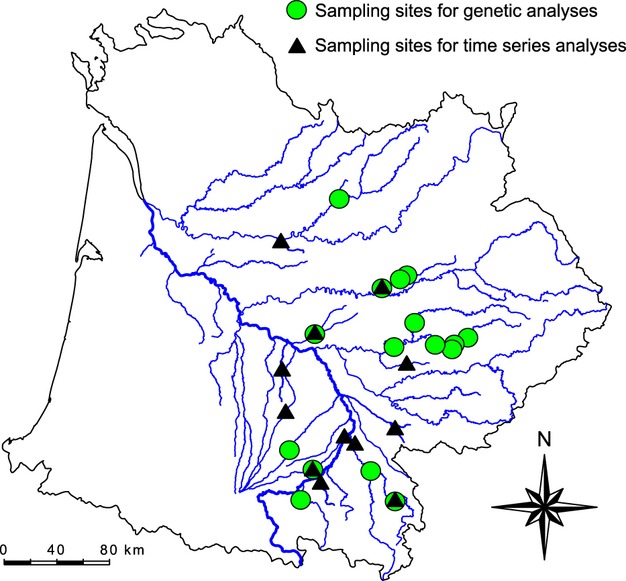
Map of the Garonne river basin (South–Western France) representing (1) sites where *Parachondrostoma toxostoma* was sampled for the genetic analyses (green circles) and (2) sites that have been selected for analyses of temporal trends in population abundances (black triangles).

Ninety-two sampling sites belonging to 34 rivers of the Garonne river basin were investigated using electrofishing in 2010 and 2011 (Fig. S1). We did not catch *P. toxostoma* at 76 sites. Two hundred and 30 individuals of *P. toxostoma* were sampled at sixteen sites (Table 1, Fig. 1). Thus, we assume that these sixteen sites are representative of the current *P. toxostoma* populations. However, due to the low numbers of individuals captured at some sampling sites, individuals from sites belonging to the same river were pooled for subsequent analyses. All genetic analyses were therefore conducted at the river level (*n*_RIVER_ = 9). A small fragment of pelvic fin was collected and stored in 90% ethanol. Individuals were all released alive at their sampling site.

**Table 1:** *Parachondrostoma toxostoma* sampling sites information

River	Code	Location	Latitude	Longitude	PGS	*N*_(PGS)_	DMP	*Y*_(DMP)_
ARRATS	ARR	Aubiet	N 43°38′48″	E 0°46′45″	–	–	X	13
AUROUE	AUR	L'isle-Bouzon	N 43°54′32″	E 0°43′45″	–	–	X	13
AVEYRON	AVE	Feneyrols	N 44°07′52″	E 1°48′51″	X	5	–	–
		Monteils	N 44°17′09″	E 2°00′07″	X	4	–	–
ARIEGE	ARI	Vénerque	N 43°26′13″	E 1°26′15″	–	–	X	8
PETITE BARGUELONNE	BAR	Montbarla	N 44°12′34″	E 1°03′40″	X	9	X	17
CELE	CEL	Boussac	N 44°35′46″	E 1°55′02″	X	7	–	–
		Sainte Eulalie	N 44°35′36″	E 1°52′25″	X	8	–	–
		Sauliac-sur-Célé	N 44°31′09″	E 1°42′58″	X	25	X	11
COUZE	COU	Bayac	N 44°48′16″	E 0°43′45″	–	–	X	14
ELLE	ELL	Terrason-Lavilledieu	N 45°08′51″	E 1°15′37″	X	25	–	–
GARONNE	GAR	Muret	N 43°27′36″	E 1°19′52″	–	–	X	10
HERS	HER	Besset	N 43°05′03″	E 1°50′24″	X	4	X	10
		Calmont	N 43°17′10″	E 1°37′59″	X	25	–	–
LOUGE	LOU	Fousseret	N 43°16′27″	E 1°04′07″	X	8	X	13
SALAT	SAL	Touille	N 43°04′38″	E 0°58′05″	X	25	–	–
SAVE	SAV	Espaon	N 43°25′20″	E 0°51′21″	X	18	–	–
VENDINELLE	VEN	La Salvetat Lauragais	N 43°32′22″	E 1°48′15″	–	–	X	18
VERE	VER	Cahuzac-sur-Vère	N 43°59′12″	E 1°53′43″	–	–	X	17
VIAUR	VIA	La Calquière	N 44°09′12″	E 2°12′15″	X	13	–	–
		Saint Just	N 44°07′24″	E 2°21′57″	X	23	–	–
		Navech	N 44°09′25″	E 2°23′18″	X	25	–	–
		Serres	N 44°12′29″	E 2°31′25″	X	6	–	–
VOLP	VOL	Plan	N 43°10′16″	E 1°07′07″	–	–	X	8

PGS (for Point Genetic Study) indicates whether the site has (X) or not (–) been sampled for genetic analyses. *N*_(PGS)_ indicates the number of individuals sampled per site for genetic analyses. DMP (for Demographic Monitoring Program) indicates whether the site has (X) or not (–) been selected for analyses of temporal trends in abundance. *Y*_(DMP)_ indicates the number of years considered in the time series.

#### Genotyping

We used a salt-extraction protocol to extract genomic DNA from pelvic fins (Aljanabi and Martinez 1). Fifteen microsatellite loci previously developed and/or evaluated for *P. toxostoma* (Dubut et al. 24) were coamplified using two multiplexed polymerase chain reactions (PCRs; see Table S1 for details on loci and primers concentrations). PCR amplifications were performed with 5–20 ng of genomic DNA and using the QIAGEN^®^ Multiplex PCR Kit (Qiagen, Valencia, CA). PCRs were carried out under conditions described by Dubut et al. (24). Genotyping was performed on an ABI PRISM^™^ 3730 Automated Capillary Sequencer (Applied Biosystems, Foster City, CA) at the “Génopole Toulouse Midi-Pyrénées” (France). Allele sizes were scored using the software GENEMAPPER^®^ v.4.0 (Applied Biosystems).

#### Descriptive genetic analyses

The presence/absence of large allele dropouts and null alleles was determined using the software MICRO-CHECKER 2.3 (Van Oosterhout et al. 86). Departures from Hardy–Weinberg (HW) equilibrium were estimated using the program GENEPOP v4.0 (Rousset 76). Levels of significance for HW were adjusted using the false discovery rate (FDR) procedure (Benjamini and Hochberg 5). Linkage disequilibrium among loci within sites was tested with the program FSTAT 2.9.3.2 (Goudet 38).

The mean number of alleles per site, the average observed (*H*_o_) and expected (*H*_e_) heterozygosity over loci, as well as *H*_o_ and *H*_e_ per loci per site were estimated using ARLEQUIN 3.5 (Excoffier and Lischer 29). We used a rarefaction procedure, as implemented in the software ADZE 1.0 (Szpiech et al. 85), to estimate allelic richness (Petit et al. 68) for each site, considering minimum sample sizes of *N* = 8 and *N* = 18 individuals.

#### Population structure

A Bayesian model-based clustering approach was used to search for the occurrence of independent genetic groups (i.e., clusters, *K*) in our dataset (as implemented in STRUCTURE 2.3.3; Pritchard et al. 72; Falush et al. 31, 32; Hubisz et al. 45). The burn-in length of the Markov Chain Monte Carlo (MCMC) was set to 50,000 followed by 200,000 iterations. The admixture model and the correlated allele frequencies model were used with priors on population sampling location (Hubisz et al. 45). Ten runs were conducted for each *K* value, with *K* ranging from 1 to 10. We used CORRSIEVE 1.6.2 (Campana et al. 11) to combine two approaches aiming at determining *K*: the Δ*K* test (Evanno et al. 27) and the Δ*F*_st_ test (Campana et al. 11).

To further assess the levels of genetic differentiation among *P. toxostoma* sites, two different indices were estimated: pairwise *F*_st_ (Weir and Hill 88) and the unbiased pairwise *D*_est_ (Jost 46), calculated using ARLEQUIN 3.5 and SMOGD (Crawford 18), respectively.

#### Demographic history inference and current *N*_e_ estimation

We used two different approaches for inferring past changes in the effective population size (i.e., expansions or contractions) of *P. toxostoma*.

The first method, implemented in the BOTTLENECK v1.2.02 software (Cornuet and Luikart 15; Piry 69), uses summary statistics of the genetic diversity to assess significant deviations from mutation/drift equilibrium. Significant heterozygosity excesses are considered as evidence of recent bottlenecks, whereas significant heterozygosity deficiencies can be interpreted as signals of recent population expansion (Luikart and Cornuet 54). We performed analyses considering two different microsatellite evolution models: the stepwise mutation model (SMM) and the two-phase model (TPM). For the latter, we set the percentage of multistep mutations at 30%. We tested the significance of mutation/drift equilibrium deviations for the two models using Wilcoxon's signed rank tests. To account for multiple comparisons, we applied the FDR procedure (Benjamini and Hochberg 5). The second method is the full-likelihood Bayesian approach implemented in the program MSVAR 1.3 (Beaumont 3; Storz and Beaumont 84). This coalescent-based method relies on a hierarchical Bayesian model to detect, date, and quantify past demographic changes. The model assumes that a stable, closed population of ancestral size *N*_1_ increased or decreased exponentially to its current size *N*_0_ (i.e., its current *N*_e_) over a time interval of *T*_a_ years. This method uses all the information contained in the data and lognormal priors to infer the parameters of the model Ф = {*N*_0_, *N*_1_, *T*_a_, θ}, where θ = 4*N*_0_ μ and μ is the mutation rate. The posterior probability density of Ф is assessed via MCMC algorithms. Microsatellite loci are assumed to be independent and to evolve under a strict SMM. For each river-scale analysis, we performed four independent runs of 5 × 10^9^ steps, considering different starting values and means for priors and hyperpriors for each run (Goossens et al. 37). We set a generation time of 3 years for *P. toxostoma* (Keith et al. 48). Parameters were thinned with an interval of 5 × 10^4^ steps, resulting in output files with 1 × 10^5^ values. We discarded the first 10% of the chains as burn-in to prevent bias induced by the starting values on parameter estimation. The convergence of the MCMC chains was checked with the Gelman and Rubin analysis implemented in the R package CODA (Gelman and Rubin 35; Plummer et al. 70). For each analysis, posterior parameter values obtained by the four independent runs were pooled together and subsequently used to calculate the median and the 5–95% quartiles for *N*_0_, *N*_1,_ and *T*_a_. We also calculated these statistics for the ratio log_10_(*N*_0_/*N*_1_). Negative values of this ratio indicate that the population has experienced a decrease in effective population size, while positive values characterize demographic expansions. This approach was also used to estimate a current *N*_e_ at the Garonne river basin scale. To do so, we ran MSVAR by pooling all individuals from all rivers in a single analysis. At such a scale, estimates of current *N*_e_ were compared to those estimated using the linkage disequilibrium-based approach implemented in LDNe (Waples and Do 87). LDNe was not used at the river scale due to its propensity to give negative *N*_e_ estimates (which are interpreted as infinity estimates, Waples and Do 87) for most rivers. MSVAR 1.3 runs were performed on an ALTIX ICE 8200 EX and UV computer cluster (Silicon Graphics International, Fremont, CA) hosted by the CALMIP group at the University Paul Sabatier (Toulouse, France).

### Demographic monitoring data

#### Database description

We used the surveillance monitoring database of the French National Agency for Water and Aquatic Environments (i.e., ONEMA) to carry out demographic trend and species distribution analyses. This database includes an extensive spatiotemporal set of monitoring surveys of French freshwater fish populations, representative of all fish assemblages and covering varying degrees of anthropogenic disturbances (Poulet et al. 71). Surveys were conducted according to standard electrofishing procedures (Poulet et al. 71). We used this database to (1) identify temporal trends in population abundance of *P. toxostoma* at 12 sampling locations; (2) assess the current spatial distribution of this species in the Garonne river basin; and (3) investigate whether the spatial distribution of this species in the Garonne river basin has declined or expanded over the last three decades.

#### Temporal trends in abundance

From this dataset, we selected all sites belonging to the Garonne river basin that have been sampled and investigated for *P. toxostoma* abundance for at least 8 years. This resulted in the selection of twelve sites (Table 1, Fig. 1) for which time series ranged between 8 and 18 years and occurred between 1991 and 2010. As sampling procedures were standardized over years, abundances (expressed as the number of individuals per m^2^) were directly comparable across years. It is noteworthy that (1) this database and the genetic database have been gathered during independent research projects; and (2) *P. toxostoma* is relatively rare in this area (Fig. S1), which both explain why demographic and genetic data are not available for all sites (see Table 1). Some sites for which long-term demographic data were available have been unsuccessfully sampled for genetic, and inversely, some sites where genetic data were available had time series that were not long enough to be analyzed (i.e., <8 years).

First, we assessed the strength and significance of temporal trends at these sites, by using a modified Mann–Kendall trend test that we independently applied to each time series (Hamed and Rao 43). In this test, the Mann–Kendall's *S* statistic (Kendall 49) provide an estimate of the strength of the association between time and the response variable, while accounting for temporal autocorrelation present in a time series (Hamed and Rao 43).

Second, we assessed whether or not these twelve time series showed an overall significant trend. For this purpose, we performed a meta-analysis (Gurevitch and Hedges 42) on the twelve Mann–Kendall's trend statistics *S* calculated in the first step. We applied a mixed linear model approach using maximum likelihood, in which we assumed that the 12 time series included in the meta-analysis share a common effect size with a random variation among the twelve time series.

#### Current spatial distribution and recent distribution changes

We used the database described above to assess changes in the spatial distribution of *P. toxostoma* on the Garonne river basin over two distinct periods, separated by a time span of 10 years (i.e., “past period”: 1980–1992, and “current period”: 2003–2009). To account for potential sampling bias when comparing spatial distributions over time based on datasets not originally collected for this purpose (Shaffer et al. 80; Shoo et al. 81), we modeled the spatial distribution of the species across the French hydrographic network as a function of several climatic and environmental variables.

Accurately modeling species distribution requires performing analyses at the entire species range scale, so as to encompass all environmental conditions (Austin 2). Therefore, for both time periods, initial models were calibrated at the French scale. We selected 3549 sites sampled over the 1980–1992 period and 3543 sites sampled over the 2003–2009 period scattered across France (see Fig. S2). The occurrence of the species was modeled independently for both time periods as a function of habitat and climatic data strongly related to fish spatial distributions (Buisson et al. 10): elevation (m), slope (%), upstream–downstream position (G), mean temperature of the coldest quarter (°C), mean temperature of the warmest quarter (°C), temperature variability, cumulated precipitations of the wettest quarter (mm), cumulated precipitations of the driest quarter (mm), and precipitation variability (Hijmans et al. 44).

To account for uncertainty in estimating species range, we used a modeling approach allowing us to produce maps of species habitat suitability (e.g., Puschendorf et al. 74; Grenouillet et al. 40). Specifically, we used an ensemble modeling approach based on a consensus model averaging the probabilities of occurrence predicted by eight single-species distribution models (Marmion et al. 58), as well as three threshold setting methods allowing the conversion of occurrence probabilities into binary data (i.e., presence or absence, Liu et al. 52), and 30 iterations (see Appendix S1 for details on models’ implementation).

The calibrated models set at the French scale were then used to predict the binary predictions of occurrence of the species for the two distinct periods in the hydrographic network of the Garonne river basin. The spatial distribution of the species for each time period was calculated as the length of the hydrographic network occupied by the species (e.g., Fagan 30) in the Garonne river basin (expressed in % of the total network length). However, because the ability to detect changes in the spatial distribution of species may be confounded by the uncertainty arising from methodological strategies (e.g., threshold effect, Nenzén and Araújo 60), temporal changes in the occupied stream length were evaluated using a linear model that controlled for the threshold effect. A linear model was thus fitted to the spatial distribution of *P. toxostoma* in both periods where the threshold-setting method and the period were used as explanatory variables. The change (i.e., extension or contraction) was then provided by the least-squares means intercepts of the contemporary period-group effect. Temporal trends analyses and spatial distribution models have been developed under the R statistical software 2.13.0 (R Development Core Team 75).

## Results

### Population genetics study

#### Descriptive genetic analyses

After applying the FDR controlling procedure, no null alleles were detected in our dataset, there were no significant deviations from HW for any loci or any population (Tables S2 and S3), and we failed to detect significant linkage disequilibrium between pairs of loci (Table S4).

**Figure 2:**
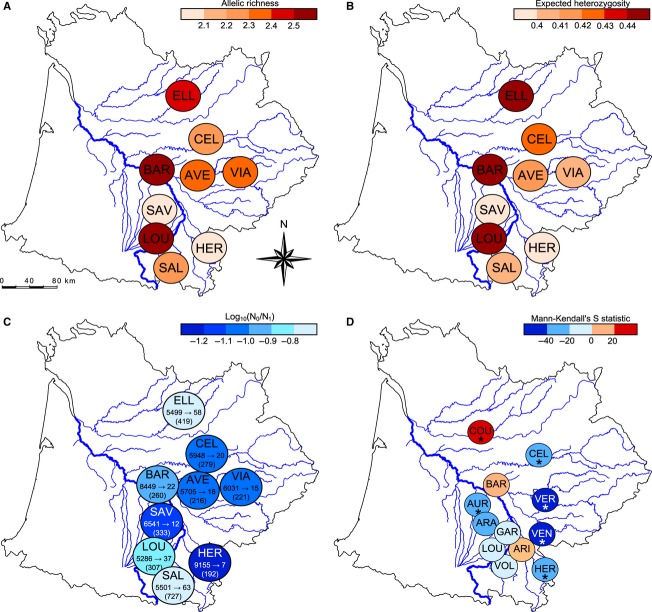
Maps representing (A) the allelic richness per population considering a minimum sample size of 8 (color scale), (B) the expected heterozygosity per population (color scale), (C) the past effective population size (*N*_1_; left number in the bubbles, see also Table S6), the current effective population size (*N*_0_; right number in the bubbles, see also Table S6), the time of the beginning of the bottlenecks (in years backward in time; numbers in brackets, see also Table S6), and the magnitude of bottlenecks (i.e., Log_10_ (*N*_0_/*N*_1_): color scale, see also Table S6), and (D) the value of the Mann–Kendall's *S* statistic (color scale) and the significance of Mann–Kendall trend tests for each time series: Asterisks (*) denote significant (i.e., *P* < 0.05) temporal trends. For all panels, the three-letter code in each bubble corresponds to the river codes (see Table 1).

Overall, genetic diversity estimates were low (Fig. 2A and B, Table S3). Loci were weakly polymorphic at the basin scale (2–6 alleles per locus), with some loci being monomorphic at the river scale (na = 1; Table S2). Average *H*_e_ and *H*_o_ values across loci within rivers were moderately low (*H*_e_ = 0.320–0.450; *H*_o_ = 0.315–0.482), as well as mean number of alleles and allelic richness estimates (AR_8_ = 1.868–2.536 alleles per river; AR_18_ = 2.147–3.037; Fig. 2A and B, Table S3). It is noteworthy that the Save River (SAV) displayed the lowest genetic diversity estimates (Fig. 2A and B, Table S3).

#### Population structure

**Figure 3:**
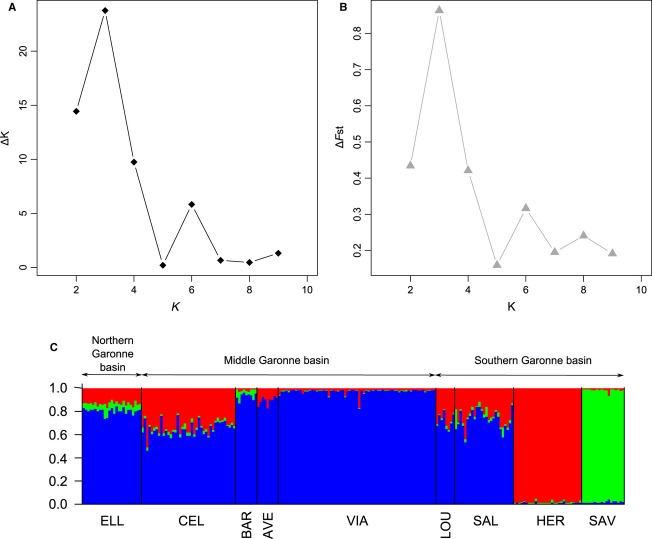
Analysis of the population structure of *Parachondrostoma toxostoma* in the Garonne river basin. (A) and (B) represent the results from *ΔK* and *ΔF*_st_ tests, respectively. (C) is a barplot representing the results of the Bayesian clustering analysis of microsatellites using STRUCTURE for *K* = 3.

The ten runs of the Bayesian clustering analysis were convergent. The *ΔK* and *ΔF*_st_ tests revealed three distinct clusters *K* = 3 (Fig. 3A–B). Most of the populations were hardly differentiable and were characterized by the occurrence of a main cluster, whose frequency range was from 62% (CEL) to 98% (VIA). Only SAV and HER were discriminated from the rest of the Garonne river basin, each site corresponding to a distinct cluster (Fig. 3C). Overall, genetic differentiation values between rivers were weak to moderate and ranged between 0.003 and 0.244 and 0.003 and 0.281 for *F*_st_ and *D*_est,_ respectively (Table 2). All but five pairwise *F*_st_ values were significant (Table 2). The stronger differentiations were found between SAV/VIA (*F*_st_ = 0.244; *D*_est_ = 0.097) and SAV/BAR (*F*_st_ = 0.117; *D*_est_ = 0.281).

**Table 2:** Population pairwise *F*_st_ (upper half-matrix) and pairwise *D*_est_ (lower half-matrix) values calculated between all rivers (denoted by their three-level code)

Code	AVE	BAR	CEL	ELL	HER	LOU	SAL	SAV	VIA
AVE	–	**0.117**	**0.067**	**0.070**	**0.042**	0.013^ns^	**0.014**	**0.109**	0.005^ns^
BAR	0.056	–	**0.102**	**0.052**	**0.054**	**0.025**	**0.026**	**0.130**	**0.017**
CEL	0.018	0.031	–	**0.023**	**0.023**	0.003^ns^	**0.012**	**0.089**	**0.010**
ELL	0.035	0.008	0.003	–	**0.032**	0.008^ns^	**0.014**	**0.115**	**0.008**
HER	0.132	0.165	0.077	0.096	–	**0.069**	**0.068**	**0.077**	**0.122**
LOU	0.054	0.057	0.029	0.029	0.019	–	0.004^ns^	**0.114**	**0.050**
SAL	0.049	0.096	0.033	0.034	0.013	0.024	–	**0.090**	**0.037**
SAV	0.262	0.281	0.221	0.230	0.241	0.265	0.228	–	**0.244**
VIA	0.026	0.093	0.044	0.034	0.033	0.015	0.010	0.097	–

For pairwise *F*_st_, significant values at level 0.05 after false discovery rate (FDR) correction are in bold. Nonsignificant pairwise *F*_st_ are denoted by ns.

#### Demographic history inference and current *N*_e_ estimation

According to the BOTTLENECK software, and after corrections for multiple tests, there was no significant evidence for demographic changes in the Garonne river basin (Table S5). On the contrary, the MSVAR analyses revealed significant signals of bottleneck in all rivers (Fig. 2C, Table S6). The magnitude of these bottlenecks, as indicated by the median values of the log_10_ (*N*_0_/*N*_1_) ratio, ranged between −0.705 (ELL) and −1.345 (HER; Fig. 2C, Table S6). Overall, *N*_0_ estimates (i.e., the current *N*_e_ of populations) were similar across rivers, with medians ranging from 7 (HER) to 63 individuals (SAL). Concerning ancestral population sizes (*N*_1_), median values ranged from 5286 (LOU) to 9155 individuals (HER; Fig. 2C, Table S6). These bottlenecks were estimated to have occurred between 192 (HER) and 727 years ago (SAL). The MSVAR method has often been considered as more powerful than the BOTTLENECK method (Williamson-Natesan 89; Girod et al. 36), which may explain the discrepancy observed between these two methods.

The analysis performed at the Garonne river scale confirmed the low estimates of current *N*_e_ found at the river scale. Indeed, at this scale, MSVAR provided an estimate of 147 individuals (5–95% quartiles: 35.6–534.4) in the whole drainage, whereas LDNe provided a global estimate of 74.6 individuals (95% CI: 54.4–104.6).

### Demographic monitoring data

#### Temporal trends in abundance

Five out of the twelve populations (i.e., HER, VEN, AUR, CEL, VER) showed a significant negative trend (*P* < 0.05; *S* < 0), one population (COU) showed a significant positive trend (*P* < 0.01; *S* = 23) whereas the remaining six populations (VOL, LOU, ARI, GAR, ARR, BAR) showed no significant trend in abundance (Fig. 2D, Table S7). Overall, the mixed model meta-analysis revealed a significant (*P* < 0.001) negative trend indicating a global decrease in the abundance of *P. toxostoma* populations in the Garonne river basin.

#### Modeling species distribution

**Figure 4:**
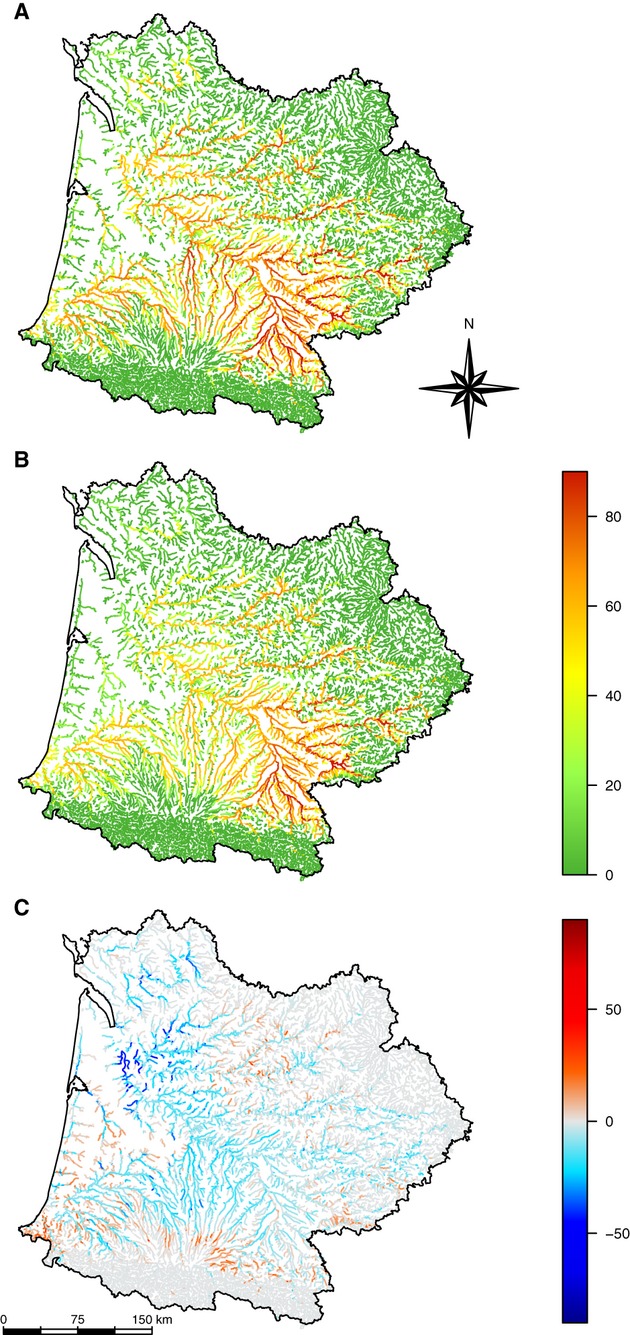
Spatial distributions of *Parachondrostoma toxostoma* modeled for (A) 1980–1992 and (B) 2003–2009 periods, and differences between these two distributions (C). The agreement between presence–absence predictions (i.e., habitat suitability) was measured by summing the 90 predictions (threshold × iteration) for each reach of the Garonne river basin for each period, with color scale varying from green (no predicted presence) to red (90 predicted presences). The differences in the spatial distribution of the species were expressed with a color scale varying from blue (90 presences predicted only for 1980–1992) to red (90 presences predicted only for 2003–2009).

**Figure 5:**
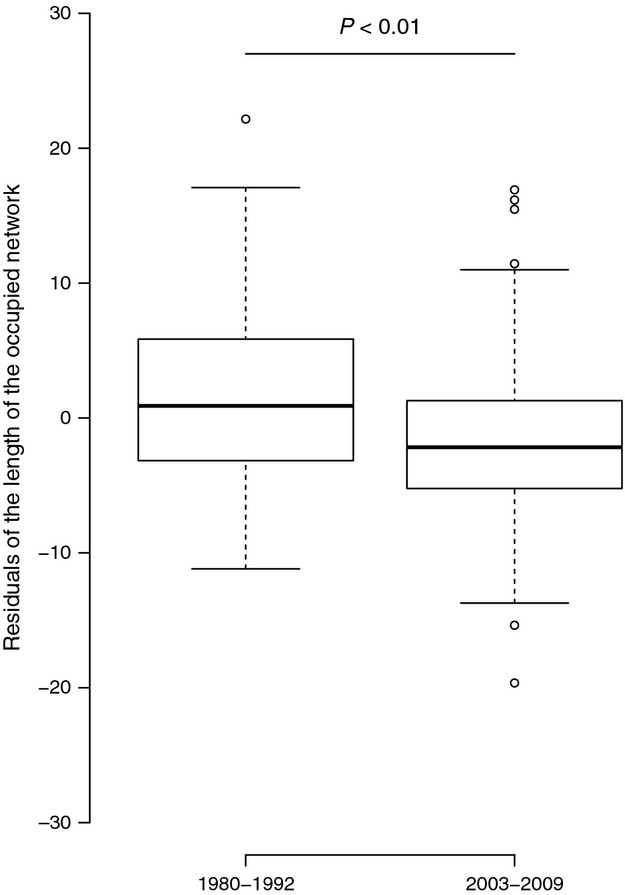
Boxplots of the length of the occupied network by *Parachondrostoma toxostoma* in the Garonne river basin modeled for the periods 1980–1992 and 2003–2009. The length of the occupied network was the residuals of a linear regression linking the length of occupied network in both periods with the threshold setting method effect.

The stream length occupied by the species was estimated at 24.0% (±2.5 SE) of the total river basin stream length in 1980–1992 (Fig. 4A) and 20.9% (±2.6 SE) in 2003–2009 (Fig. 4B). This represented an overall decrease of 3.2% (*P* < 0.01) with respect to the whole river basin, and of 13.1% of *P. toxostoma's* 1980–1992 distribution (Figs. 4C, 5). The habitat suitability for the species decreased in the middle part of the river basin between 1980 and 1992 and 2003 and 2009 periods (Fig. 4).

## Discussion

### What did we learn from genetic data?

Using a full-likelihood Bayesian approach (as implemented in MSVAR, Storz and Beaumont 84), we showed that all *P. toxostoma* populations have experienced significant decreases in effective population size (*N*_e_*)*, with reductions of more than 99% of their prebottleneck long-term *N*_e_. We further showed that: (1) in all populations, bottlenecks started 192–727 years ago, and are hence relatively recent (i.e., within the last millennium); and (2) all populations show extremely low current *N*_e_. Attempting to identify the causes of such bottlenecks would be highly speculative without further data and analyses. If natural causes (climatic or hydrological shifts) cannot be ruled out, anthropogenic causes are also likely (i.e., the first mill weirs date back from the 12th century, Blanchet et al. 7). It is noteworthy that the bottlenecks highlighted here are “species-specific” rather than “basin-specific”, given that for four other sympatric cyprinid fish species (i.e., *Squalius cephalus*,* Leuciscus burdigalensis, Gobio gobio,* and *Phoxinus phoxinus*), Paz-Vinas et al. (67) demonstrated that bottlenecks were older (approximately 2000–6000 years ago) and of different magnitudes than those detected for *P. toxostoma*. We can hence reasonably conclude that the bottlenecks inferred here occurred during the last millennium and affected specifically *P. toxostoma* populations.

Descriptive analyses revealed low levels of genetic diversity for all populations. Indeed, all diversity indices were up to approximately 3.3 times lower than those calculated for populations of other cyprinid fish species co-occurring with *P. toxostoma* in the Garonne river basin (Blanchet et al. 7). They were all also remarkably lower than those calculated for *P. toxostoma* populations from the Rhône river basin (see Dubut et al. 24). As an example, some microsatellite markers were monomorphic in certain populations, whereas these same markers were highly polymorphic in populations from the Rhône river basin (Dubut et al. 24). Similarly, Costedoat et al. (16) demonstrated that the diversity measured at mitochondrial genes for *P. toxostoma* was also significantly lower in the Garonne river basin than in the Rhône river basin, a result that may be a consequence of the recent colonization of the Garonne river basin from the Rhône river basin (i.e., approximately 57,000 years ago, Costedoat et al. 16). Although the relatively poor genetic diversity found in the Garonne river basin probably has an important phylogeographical basis (Costedoat et al. 16), it may reflect the more recent (200–700 years ago) and severe bottlenecks that we detected.

Finally, our PGS also highlighted that *P. toxostoma* populations in the Garonne river basin were relatively homogeneous from a genetic standpoint. Indeed, most populations formed a single cluster with relatively low genetic differentiation within this cluster. This result suggests that these populations constitute a single panmictic unit at the basin level. There were, however, two noticeable exceptions to this general pattern; HER and SAV were genetically differentiated from all other populations. These two populations also demonstrated the lowest contemporary *N*_e_ values, the lowest genetic diversities (i.e., *H*_e_, *H*_o,_ and AR), and the strongest bottlenecks. Altogether, this indicates that these populations may be discriminated from others (1) because gene flow between these populations and others are weak; and/or (2) because genetic drift and inbreeding were particularly high in these populations, causing divergence from other populations in the Garonne river basin.

To summarize, PGS provided a precise description of the current genetic state of *P. toxostoma* populations from the Garonne river basin. Overall, these results clearly indicate that long-term management should integrate the fact that the evolutionary potential of the species in this geographic area may be weak.

### What did we learn from demographic data?

Using time series abundance data at twelve locations, we found an overall demographic decrease of *P. toxostoma* populations that occurred in the last three decades. Evidence of a demographic decrease was further supported by comparing the *P. toxostoma* occurrence at the basin scale between two periods (1980–1992 and 2003–2009). This analysis revealed a significant decrease in the distribution range of *P. toxostoma,* representing 13.1% of the 1980–1992's distribution. These results confirm that over the range of the species, there is a decreasing trend in abundance (Crivelli 19; Poulet et al. 71). This decrease contrasts with the increase in occurrence, abundance, and density of several sympatric species at the French scale such as *Barbus barbus* or *Gobio gobio* (Daufresne and Boët 20; Poulet et al. 71). Despite this range-wide trend, we showed that not all local populations were subjected to a significant demographic decrease, as some of them display no particular trends, and one population even showed a significant demographic increase. There was no clear spatial pattern regarding these site-specific trends (see Fig. 2D). However, such site-specific analysis provides a basis for further analyses exploring the regional and/or local causes of demographic trends in the Garonne river basin. Indeed, a comparison implying healthy versus nonhealthy (from a demographic point of view) populations may highlight the leading environmental factors affecting the demography of this species.

To summarize, DMPs provided insights into the demographic dynamics and changes in the spatial distribution of *P. toxostoma* in the Garonne river basin, which indicates that this species is ecologically weakened in this area, and thus restoration plans should be engaged to ensure the persistence of populations.

### Synthesis, implications, and conclusions: The conservation gain of combining genetic and demographic data

#### Synthesis

The history of *P. toxostoma* in the Garonne river basin is relatively recent and began approximately 57,000 years ago, when it colonized the Garonne from the Rhône river basin (Costedoat et al. 16). Our results suggest that populations exhibited relatively large long-term *N*_e_ (approximately 5000–8000 individuals per population) until severe and recent (approximately 800 to 200 years ago) demographic collapses entailed *N*_e_ of less than a few hundred (sometimes less than a dozen) individuals. This means that very small numbers of effective breeders are currently sustaining populations in the Garonne river basin. This history led to genetically impoverished *P. toxostoma* populations in the Garonne river basin. Although most populations are genetically homogeneous, these demographic collapses also led to local differentiation in the Garonne river basin. In a more recent timeframe (i.e., the last two decades), we showed that this species experienced a global decrease in census size (*N*_c_) over the entire Garonne river basin, although that some populations remained demographically stable or even increased locally. This recent decrease in *N*_c_ was accompanied by a significant reduction of its spatial distribution over the Garonne river basin. Because both *N*_e_ and *N*_c_ are reduced in these populations, *P. toxostoma* in the Garonne river basin is confronted with a combination of ecological and evolutionary extinction risks, which reinforces its status of vulnerable species in the IUCN red list, and supports the implementation of conservation plans.

#### Implications

Our results illustrate how combining genetic and demographic approaches is useful to target and to prioritize conservation and management plans for endangered populations. A main weakness of our study resides in the few number of sampling points common to both temporal trend and genetic analyses. However, this fact may well be the standard for most studies focusing on rare and threatened species. We therefore provide recommendations considering two cases. In the first case, both demographic and genetic are available at the sampling site level. In this case, combining genetic and demographic approaches allows identifying priority populations as those (1) having the lowest genetic diversity and *N*_e_; and (2) being subjected to a significant and recent decrease in *N*_c_. For instance, we identified the Hers River as a priority population as both genetic and demographic indices are weak. In this case, we propose conservation strategies involving a program of stocking from broodstock stemming from healthy populations, combined with the restoration of habitat and connectivity with other rivers. Healthy populations are those with stable *N*_c_ and higher *N*_e_ (such as the Petite Barguelonne and Louge rivers). In the second case, only one of the two metrics is available at the sampling site level. In this case, prioritizing conservation plans is less straightforward. For instance, some populations (e.g., the Vendinelle River) were subjected to a sharp decrease in *N*_c_ in recent years, however, no data are yet available regarding genetic diversity and *N*_e_ dynamics. In this case, managers can conduct a genetic monitoring of these populations to help clarify the populations’ status. On the other hand, some populations (e.g., the SAV) have low *N*_e_ and low genetic diversity, but lack temporal data regarding *N*_c_. In this case, it is impossible to get the temporal trend of the populations. Thus, invoking the precautionary principle, we propose considering these populations as conservation priority.

## Conclusion

To conclude, we showed how combining analyses based on point genetic studies (PGSs) and DMPs (i.e., a “demo-genetic approach”) reveal complementary information underlying different processes operating at different timescales. Demo-genetic approaches allow (1) identification of “at risk” populations; (2) prioritizing conservation and management actions; and (3) proposing plans that account for the evolutionary history and potential of populations. We hence argue that demo-genetic approaches should be the norm in conservation practices. Indeed, these surveys would allow not only prioritizing and initiation of conservation plans (this study), but would also allow the evaluation of dispersal and connectivity through the use of genetic-based inference methods (Broquet and Petit 8), as well as evaluation of the effectiveness of conservation plans (Schwartz et al. 79; Osborne et al. 64). We hope that this study will motivate conservation ecologists to invest in genetic monitoring, and conversely, conservation geneticists to initiate long-term demographic surveys.

## Supporting Information

**Appendix S1.** Current spatial distribution and recent distribution changes. **Figure S1**. Map of the Garonne river basin (South–Western France) representing (1) sites where *P. toxostoma* was unsuccessfully sampled for genetic analyses (white circles); and (2) sites where *P. toxostoma* was successfully sampled for genetic analyses (green circles). **Figure S2.** Maps representing sites where the occurrence of *P. toxostoma* was recorded (A) from 1 to 19 times during the 1980–1992 period and (B) from 1 to 14 times during the 2003–2009 period. **Table S1.** Information on microsatellite loci and multiplexed PCR used in this study **Table S2.** Observed number of alleles (na), expected (*H*_e_) and observed (*H*_o_) heterozygosities and departures from Hardy–Weinberg equilibrium (F*is*) for all loci and populations of *P. toxostoma*. No significant departures from Hardy–Weinberg equilibrium were found after applying Benjamini and Hochberg (1995) false discovery rate corrections. **Table S3.** Mean observed (*H*_o_) and expected (*H*_e_) heterozygosities, mean number of alleles over loci (NA), allelic richness (AR_8_ for a minimum sample size of 8 individuals; AR_18_ for a minimum sample size of 18 individuals) and departures from Hardy–Weinberg equilibrium (*F*_is_) for each *P. toxostoma* population. **Table S4. ***P*-values for the linkage disequilibrium test for each pair of loci and population. **Table S5.** Results for the Wilcoxon's sign rank test computed by BOTTLENECK for each river and for the TPM and SMM microsatellite mutation models. **Table S6.** Median, 5 and 95% quartile values calculated for *N*_0_ (the current effective population size), *N*_1_ (the past effective population size), *T*_a_ (the time of the beginning of the demographic change, in years backwards from the present) and Log_10_ (*N*_0_/*N*_1_; the magnitude of the demographic change) for each river, through the posterior distributions obtained with MSVAR 1.3. **Table S7.** Values for the Mann–Kendall's *S* statistic, variance in S (Var[S]), mean densities and *P*-values obtained for the twelve time series with the modified Mann–Kendall trend test.
