# Supplementary material for: Combining genetic and demographic data for prioritizing conservation actions: insights from a threatened fish species
Source: Ecol Evol. 2013 Jul 9;3(8):2696–710. doi: 10.1002/ece3.645 (PMC3930054; doi:10.1002/ece3.645)
Supplement: Supplementary file 1 — Appendix S1. Current spatial distribution and recent distribution changes. Figure S1. Map of the Garonne river basin (South–Western France) representing (1) sites where P. toxostoma was unsuccessfully sampled for genetic analyses (white circles); and (2) sites where P. toxostoma was successfully sampled for genetic analyses (green circles). Figure S2. Maps representing sites where the occurrence of P. toxostoma was recorded (A) from 1 to 19 times during the 1980–1992 period and (B) from 1 to 14 times during the 2003–2009 period. Table S1. Information on microsatellite loci and multiplexed PCR used in this study Table S2. Observed number of alleles (na), expected (He) and observed (Ho) heterozygosities and departures from Hardy–Weinberg equilibrium (Fis) for all loci and populations of P. toxostoma. No significant departures from Hardy–Weinberg equilibrium were found after applying Benjamini and Hochberg (1995) false discovery rate corrections. Table S3. Mean observed (Ho) and expected (He) heterozygosities, mean number of alleles over loci (NA), allelic richness (AR8 for a minimum sample size of 8 individuals; AR18 for a minimum sample size of 18 individuals) and departures from Hardy–Weinberg equilibrium (Fis) for each P. toxostoma population. Table S4. P-values for the linkage disequilibrium test for each pair of loci and population. Table S5. Results for the Wilcoxon's sign rank test computed by BOTTLENECK for each river and for the TPM and SMM microsatellite mutation models. Table S6. Median, 5 and 95% quartile values calculated for N0 (the current effective population size), N1 (the past effective population size), Ta (the time of the beginning of the demographic change, in years backwards from the present) and Log10 (N0/N1; the magnitude of the demographic change) for each river, through the posterior distributions obtained with MSVAR 1.3. Table S7. Values for the Mann–Kendall's S statistic, variance in S (Var[S]), mean densities and P-values obtained for the [file ece30003-2696-SD1.doc]

**Appendix S1:** *Current spatial distribution and recent distribution changes*

The occurrence of *P. toxostoma* was recorded at each site from 1 to 19 times during the 1980-1992 period and from 1 to 14 times during the 2003-2009 period, resulting in 4533 and 7548 sampling occasion records, respectively (see Figure S1).

Independent models were then implemented to model the occurrence of the species in each time period based on a single sampling occasion records (i.e., presence or absence) randomly chosen for each site. We used a ensemble modeling approach allowing to account for variability associated to methodological choices and data quality (e.g., Puschendorf et al. 2009; Grenouillet et al. 2011).

Specifically, to account for the variability related to the modeling method, we followed the procedure applied in Marmion et al. (2009) by averaging the probabilities of occurrence predicted by eight single-species distribution models: Generalized Linear Models (GLM), Generalized Additive Models (GAM), Multivariate Adaptive Regression Splines (MARS), Mixture Discriminant Analyses (MDA), Classification And Regression Trees (CART), Random Forest (RF), Generalized Boosted Trees (GBT) and Artificial Neural Networks (ANN).

Models were calibrated on 70% of the sampling occasion records, while the remaining 30% were used for evaluation and threshold selection. Three common threshold-setting methods were then applied to find the thresholds that offer the best trade-off to convert occurrence probabilities into binary data (i.e., presence or absence, Liu et al., 2005). Specifically, we used threshold values maximizing the sum of sensitivity and specificity, sensitivity equaling specificity and maximizing Kappa. Finally, we accounted for the variability due to the quality of the calibration dataset by repeating 30 times with 30 different sampling occasion datasets the different steps of the modeling process.

Therefore, we ultimately obtained 90 final modeled species distributions for each period resulting from 30 iterations, and 3 thresholds that we used to estimate the extent of the spatial distribution of *P. toxostoma* in both time periods.

**Literature cited**

Grenouillet, G., L. Buisson, N. Casajus, and S. Lek. 2011. Ensemble modelling of species distribution: the effects of geographical and environmental ranges. Ecography **34**:9–17.

Liu, C., P.M. Berry, T.P. Dawson, and R.G. Pearson. 2005. Selecting thresholds of occurrence in the prediction of species distributions. Ecography **28**:385–393.

Marmion, M., M. Parviainen, M. Luoto, R.K. Heikkinen, and W. Thuiller. 2009. Evaluation of consensus methods in predictive species distribution modelling. m

Puschendorf, R., A.C. Carnaval, J. VanDerWal, H. Zumbado-Ulate, G. Chaves, F. Bolaños, and R.A. Alford. 2009. Distribution models for the amphibian chytrid *Batrachochytrium dendrobatidis* in Costa Rica: proposing climatic refuges as a conservation tool. Diversity and Distributions **15**:401–408.

**Figure S1**: Map of the Garonne river basin (South-Western France) representing (i) sites where *P. toxostoma* was unsuccessfully sampled for genetic analyses (white circles) and (ii) sites where *P. toxostoma* was successfully sampled for genetic analyses (green circles).


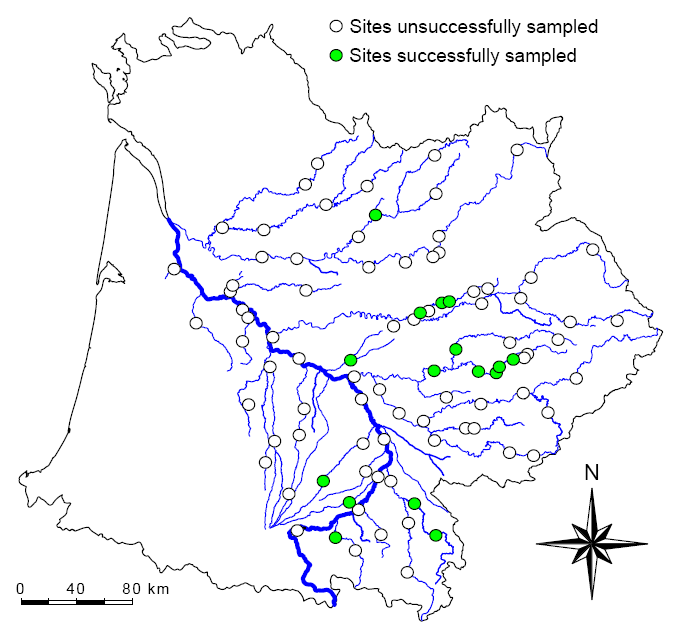


**Figure S2**: Maps representing sites where the occurrence of *P. toxostoma* was recorded (A) from 1 to 19 times during the 1980-1992 period and (B) from 1 to 14 times during the 2003-2009 period.

**
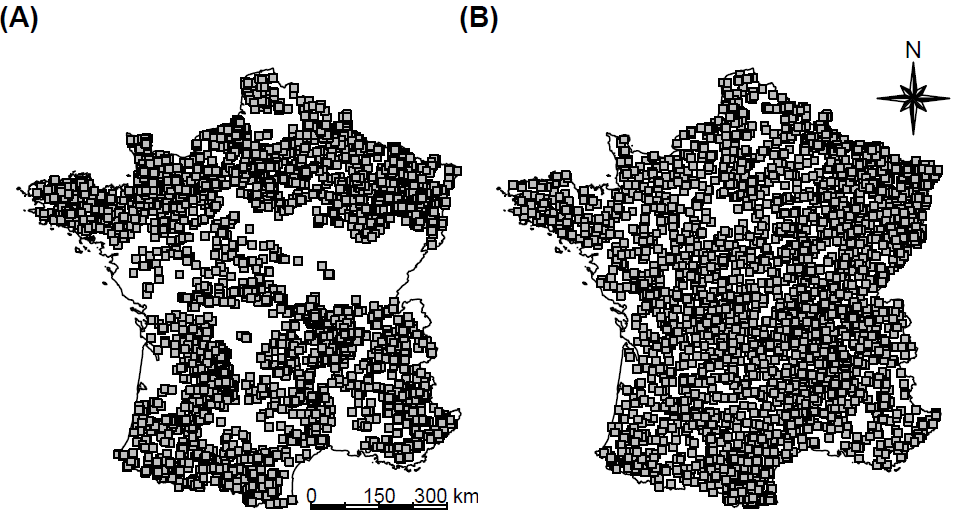
**

**Table S1:** Information on microsatellite loci and multiplexed PCR used in this study.

| Locus | GenBank Accession ID | Ref. | Fluorescent dye | Multiplex Kit | Primers concentration |
| --- | --- | --- | --- | --- | --- |
| BL1-30 | FJ468353 | Dubut et al. (2009a) | 6-FAM | 1 | 100 nM |
| Rser10 | AJ312850 | Dawson et al. (2003) | 6-FAM | 1 | 100 nM |
| LleC-090 | FJ601722 | Dubut et al. (2009b) | 6-FAM | 1 | 100 nM |
| LceC1 | AY962241 | Larno et al. (2005) | HEX | 1 | 100 nM |
| BL1-61 | FJ468351 | Dubut et al. (2009a) | HEX | 1 | 75 nM |
| Ca1 | AF277573 | Dimsoski et al. (2000) | ATTO550 | 1 | 300 nM |
| N7K4 | AJ566138 | Mesquita et al. (2003) | ATTO550 | 1 | 100 nM |
| CtoA-256 | GU254032 | Dubut et al. (2010) | 6-FAM | 2 | 150 nM |
| BL1-2b | FJ468347 | Dubut et al. (2009a) | 6-FAM | 2 | 100 nM |
| Rru4 | AB112740 | Barinova et al. (2004) | 6-FAM | 2 | 100 nM |
| Lsou34 | EF209012 | Muenzel et al. (2007) | HEX | 2 | 75 nM |
| LleA-029 | FJ601714 | Dubut et al. (2009b) | HEX | 2 | 100 nM |
| CtoF-172 | GU254034 | Dubut et al. (2010) | ATTO550 | 2 | 100 nM |
| Lsou05 | EF209002 | Muenzel et al. (2007) | ATTO550 | 2 | 100 nM |
| BL1-T2 | FJ468348 | Dubut et al. (2009a) | ATTO550 | 2 | 100 nM |

**Literature cited**

Barinova A., E. Yadrenkina, M. Nakajima, and N. Taniguchi, 2004. Identification and characterization of microsatellite DNA markers developed in ide *Leuciscus idus* and Siberian roach *Rutilus rutilus*. Molecular Ecology Notes **4**:86-88.

Dawson D.A., T.M. Burland, A. Douglas, S.C. Le Comber, and M. Bradshaw. 2003. Isolation of microsatellite loci in the freshwater fish, the bitterling *Rhodeus sericeus* (Teleostei: Cyprinidae). Molecular Ecology Notes **3**:199-202.

Dimsoski P., G.P. Toth, and M.J. Bagley. 2000. Microsatellite characterization in central stoneroller *Campostoma anomalum* (Pisces: Cyprinidae). Molecular Ecology **9**:2187-2189.

Dubut V., J.F. Martin, C. Costedoat, R. Chappaz, and A. Gilles. 2009a. Isolation and characterization of polymorphic microsatellite loci in the freshwater fishes *Telestes souffia* and *Telestes muticellus* (Teleostei: Cyprinidae). Molecular Ecology Resources **9**:1001-1005.

Dubut V., J.F. Martin, A. Gilles, J. Van Houdt, R. Chappaz, and C. Costedoat. 2009b. Isolation and characterization of polymorphic microsatellite loci for the dace complex: *Leuciscus leuciscus* (Teleostei: Cyprinidae). Molecular Ecology Resources **9**:1179-1183.

Dubut V., M. Sinama, J.F. Martin, E. Meglécz, J. Fernandez, R. Chappaz, *et al*. 2010. Cross-species amplification of 41 microsatellites in European cyprinids: A tool for evolutionary, population genetics and hybridization studies. BMC Research Notes **3**: 135.

Larno V., S. Launey, A. Devaux, and J. Laroche. 2005. Isolation and characterization of microsatellite loci from chub *Leuciscus cephalus* (Pisces: Cyprinidae). Molecular Ecology Notes **5**:752-754.

Mesquita N., C. Cunha, B. Hänfling, G.R. Carvalho, L. Zé-Zé, R. Tenreiro, *et al*. 2003. Isolation and characterization of polymorphic microsatellite loci in the endangered Portuguese freshwater *Squalius aradensis* (Cyprinidae). Molecular Ecology Notes **3**:572-574.

Muenzel F.M., M. Sanetra, W. Salzburger, and A. Meyer. 2007. Microsatellites from the vairone *Leuciscus souffia* (Pisces: Cyprinidae) and their application to closely related species. Molecular Ecology Notes **7**:1048-1050.

**Table S2:** Observed number of alleles (*na*), expected (H_e_) and observed (H_o_) heterozygosities and departures from Hardy-Weinberg equilibrium (F*is*) for all loci and populations of *P. toxostoma*. No significant departures from Hardy-Weinberg equilibrium were found after applying Benjamini and Hochberg (1995) false discovery rate corrections. N indicates the sample size.

|  | Code | AVE | BAR | CEL | ELL | HER | LOU | SAL | SAV | VIA |
| --- | --- | --- | --- | --- | --- | --- | --- | --- | --- | --- |
| Locus | N | 9 | 9 | 40 | 25 | 29 | 8 | 25 | 18 | 67 |
| BL1-30 | *na* | 3 | 3 | 3 | 4 | 3 | 4 | 3 | 2 | 3 |
|  | *He* | 0.370 | 0.426 | 0.266 | 0.316 | 0.451 | 0.328 | 0.150 | 0.486 | 0.293 |
|  | *Ho* | 0.444 | 0.333 | 0.275 | 0.360 | 0.552 | 0.375 | 0.120 | 0.500 | 0.254 |
|  | *Fis* | -0.143 | 0.273 | -0.021 | -0.119 | -0.206 | -0.077 | 0.217 | 0 | 0.141 |
| BL1-61 | *na* | 3 | 2 | 3 | 3 | 3 | 4 | 4 | 4 | 3 |
|  | *He* | 0.364 | 0.278 | 0.464 | 0.442 | 0.216 | 0.328 | 0.314 | 0.542 | 0.343 |
|  | *Ho* | 0.444 | 0.333 | 0.500 | 0.480 | 0.241 | 0.375 | 0.280 | 0.667 | 0.254 |
|  | *Fis* | -0.164 | -0.143 | -0.066 | -0.065 | -0.101 | -0.077 | 0.130 | -0.204 | 0.268 |
| LleC-090 | *na* | 6 | 6 | 4 | 6 | 3 | 4 | 5 | 4 | 6 |
|  | *He* | 0.716 | 0.531 | 0.502 | 0.571 | 0.267 | 0.539 | 0.610 | 0.497 | 0.685 |
|  | *Ho* | 0.778 | 0.667 | 0.500 | 0.560 | 0.310 | 0.625 | 0.640 | 0.389 | 0.657 |
|  | *Fis* | -0.028 | -0.200 | 0.017 | 0.040 | -0.146 | -0.094 | -0.030 | 0.244 | 0.049 |
| LceC1 | *na* | 2 | 2 | 2 | 2 | 3 | 2 | 2 | 2 | 2 |
|  | *He* | 0.346 | 0.346 | 0.469 | 0.480 | 0.492 | 0.375 | 0.493 | 0.494 | 0.468 |
|  | *Ho* | 0.444 | 0.222 | 0.600 | 0.400 | 0.586 | 0.500 | 0.560 | 0.444 | 0.328 |
|  | *Fis* | -0.231 | 0.407 | -0.268 | 0.186 | -0.175 | -0.273 | -0.116 | 0.128 | 0.305 |
| Ca1 | *na* | 4 | 6 | 6 | 6 | 4 | 5 | 5 | 3 | 6 |
|  | *He* | 0.599 | 0.790 | 0.748 | 0.781 | 0.662 | 0.633 | 0.723 | 0.370 | 0.773 |
|  | *Ho* | 0.667 | 0.778 | 0.750 | 0.600 | 0.690 | 0.625 | 0.600 | 0.222 | 0.761 |
|  | *Fis* | -0.055 | 0.074 | 0.010 | 0.251 | -0.025 | 0.079 | 0.190 | 0.424 | 0.023 |
| Rser10 | *na* | 2 | 2 | 2 | 2 | 2 | 2 | 2 | 2 | 2 |
|  | *He* | 0.475 | 0.346 | 0.455 | 0.295 | 0.498 | 0.469 | 0.480 | 0.494 | 0.322 |
|  | *Ho* | 0.556 | 0.444 | 0.600 | 0.280 | 0.586 | 0.250 | 0.480 | 0.556 | 0.373 |
|  | *Fis* | -0.111 | -0.231 | -0.307 | 0.072 | -0.161 | 0.517 | 0.020 | -0.097 | -0.152 |
| N7K4 | *na* | 5 | 5 | 6 | 6 | 4 | 4 | 6 | 3 | 5 |
|  | *He* | 0.667 | 0.654 | 0.740 | 0.757 | 0.526 | 0.734 | 0.798 | 0.286 | 0.712 |
|  | *Ho* | 0.556 | 0.889 | 0.700 | 0.800 | 0.586 | 0.750 | 0.840 | 0.222 | 0.746 |
|  | *Fis* | 0.223 | -0.306 | 0.066 | -0.037 | -0.097 | 0.046 | -0.033 | 0.249 | -0.041 |
| Rru4 | *na* | 3 | 2 | 3 | 5 | 2 | 3 | 4 | 3 | 4 |
|  | *He* | 0.290 | 0.346 | 0.496 | 0.546 | 0.384 | 0.625 | 0.253 | 0.249 | 0.510 |
|  | *Ho* | 0.111 | 0 | 0.525 | 0.680 | 0.241 | 0.625 | 0.280 | 0.167 | 0.463 |
|  | *Fis* | 0.652 | 1 | -0.045 | -0.225 | 0.386 | 0.067 | -0.087 | 0.354 | 0.100 |
| Lsou34 | *na* | 1 | 2 | 2 | 2 | 2 | 2 | 1 | 1 | 1 |
|  | *He* | 0 | 0.494 | 0.049 | 0.180 | 0.034 | 0.117 | 0 | 0 | 0 |
|  | *Ho* | 0 | 0.444 | 0.050 | 0.200 | 0.035 | 0.125 | 0 | 0 | 0 |
|  | *Fis* | NA | 0.158 | -0.013 | -0.091 | NA | NA | NA | NA | NA |
| Lsou05 | *na* | 4 | 4 | 4 | 5 | 2 | 3 | 4 | 2 | 4 |
|  | *He* | 0.624 | 0.593 | 0.434 | 0.663 | 0.400 | 0.477 | 0.545 | 0.153 | 0.569 |
|  | *Ho* | 0.889 | 0.778 | 0.500 | 0.600 | 0.414 | 0.500 | 0.560 | 0.167 | 0.582 |
|  | *Fis* | -0.376 | -0.258 | -0.139 | 0.116 | -0.018 | 0.018 | -0.008 | -0.063 | -0.016 |
| LleA-029 | *na* | 4 | 4 | 6 | 5 | 6 | 6 | 6 | 2 | 5 |
|  | *He* | 0.568 | 0.642 | 0.591 | 0.568 | 0.710 | 0.734 | 0.663 | 0.475 | 0.399 |
|  | *Ho* | 0.667 | 0.778 | 0.550 | 0.600 | 0.759 | 0.875 | 0.600 | 0.667 | 0.388 |
|  | *Fis* | -0.116 | -0.155 | 0.081 | -0.036 | -0.051 | -0.126 | 0.116 | -0.378 | 0.036 |
| CtoF-172 | *na* | 1 | 1 | 2 | 1 | 1 | 1 | 1 | 1 | 1 |
|  | *He* | 0.568 | 0.642 | 0.591 | 0.568 | 0.710 | 0.734 | 0.663 | 0.475 | 0.399 |
|  | *Ho* | 0.667 | 0.778 | 0.550 | 0.600 | 0.759 | 0.875 | 0.600 | 0.667 | 0.388 |
|  | *Fis* | NA | NA | NA | NA | NA | NA | NA | NA | NA |
| CtoA-256 | *na* | 2 | 2 | 2 | 2 | 2 | 2 | 2 | 2 | 2 |
|  | *He* | 0.475 | 0.401 | 0.500 | 0.471 | 0.462 | 0.469 | 0.497 | 0.198 | 0.486 |
|  | *Ho* | 0.333 | 0.556 | 0.550 | 0.600 | 0.379 | 0.250 | 0.360 | 0.222 | 0.409 |
|  | *Fis* | 0.351 | -0.333 | -0.088 | -0.254 | 0.196 | 0.517 | 0.294 | -0.097 | 0.166 |
| BL1-T2 | *na* | 3 | 4 | 3 | 4 | 2 | 2 | 2 | 3 | 3 |
|  | *He* | 0.426 | 0.624 | 0.528 | 0.566 | 0.366 | 0.490 | 0.385 | 0.554 | 0.395 |
|  | *Ho* | 0.556 | 0.667 | 0.525 | 0.600 | 0.276 | 0.286 | 0.280 | 0.500 | 0.388 |
|  | *Fis* | -0.250 | -0.011 | 0.019 | -0.041 | 0.263 | 0.478 | 0.291 | 0.126 | 0.025 |
| BL1-2b | *na* | 1 | 2 | 1 | 2 | 1 | 2 | 2 | 1 | 2 |
|  | *He* | 0 | 0.278 | 0 | 0.039 | 0 | 0.117 | 0.077 | 0 | 0.138 |
|  | *Ho* | 0 | 0.333 | 0 | 0.040 | 0 | 0.125 | 0.080 | 0 | 0.149 |
|  | *Fis* | NA | -0.143 | NA | NA | NA | NA | -0.021 | NA | -0.073 |

**Table S3:** Mean observed (H_o_) and expected (H_e_) heterozygosities, mean number of alleles over loci (NA), allelic richness (AR_8_ for a minimum sample size of 8 individuals; AR_18_ for a minimum sample size of 18 individuals) and departures from Hardy-Weinberg equilibrium (F*is*) for each *P. toxostoma* population. Numbers inside brackets represent the standard deviation for H_o_ and H_e_*.* N indicates the sample size.

^ns^ = non-significant departure from Hardy-Weinberg proportions

| Code | N | H_o_ | H_e_ | NA | AR_8_ | AR_18_ | F*is* |
| --- | --- | --- | --- | --- | --- | --- | --- |
| AVE | 9 | 0.395 (0.239) | 0.418 (0.253) | 2.933 | 2.375 | − | -0.030 ^ns^ |
| BAR | 9 | 0.450 (0.198) | 0.476 (0.210) | 3.133 | 2.536 | − | -0.012 ^ns^ |
| CEL | 40 | 0.418 (0.234) | 0.423 (0.237) | 3.267 | 2.289 | 2.712 | -0.049 ^ns^ |
| ELL | 25 | 0.445 (0.237) | 0.454 (0.242) | 3.667 | 2.466 | 3.037 | 0.002 ^ns^ |
| HER | 29 | 0.364 (0.223) | 0.371 (0.226) | 2.667 | 2.028 | 2.306 | -0.017 ^ns^ |
| LOU | 8 | 0.429 (0.222) | 0.458 (0.237) | 3.067 | 2.501 | − | 0.087 ^ns^ |
| SAL | 25 | 0.399 (0.259) | 0.407 (0.264) | 3.267 | 2.294 | 2.798 | 0.072 ^ns^ |
| SAV | 18 | 0.320 (0.208) | 0.329 (0.214) | 2.333 | 1.868 | 2.141 | 0.044 ^ns^ |
| VIA | 67 | 0.406 (0.236) | 0.409 (0.238) | 3.267 | 2.307 | 2.751 | 0.063 ^ns^ |

**Table S4:** P-values for the linkage disequilibrium test for each pair of loci and population. The adjusted significance threshold value for 5% nominal level is α = 0.000048.

| Locus1 | Locus2 | AVE | BAR | CEL | ELL | HER | LOU | SAL | SAV | VIA | All |
| --- | --- | --- | --- | --- | --- | --- | --- | --- | --- | --- | --- |
| BL1-30 | BL1-61 | 0.842 | 0.024 | 0.922 | 0.715 | 0.719 | 0.287 | 1.000 | 0.571 | 0.369 | 0.599 |
| BL1-30 | LleC-0 | 1.000 | 0.818 | 0.007 | 0.766 | 0.026 | 0.681 | 0.889 | 0.905 | 0.349 | 0.155 |
| BL1-30 | LceC1 | 0.365 | 1.000 | 0.636 | 0.227 | 0.864 | 1.000 | 1.000 | 0.166 | 0.633 | 0.768 |
| BL1-30 | Ca1 | 0.935 | 1.000 | 0.410 | 0.396 | 0.755 | 0.179 | 0.142 | 0.185 | 0.925 | 0.493 |
| BL1-30 | Rser10 | 0.844 | 1.000 | 0.707 | 0.535 | 1.000 | 0.504 | 0.019 | 0.642 | 0.906 | 0.900 |
| BL1-30 | N7K4 | 1.000 | 0.612 | 0.975 | 0.857 | 0.977 | 1.000 | 0.226 | 0.216 | 0.267 | 0.885 |
| BL1-30 | Rru4 | 1.000 | 0.054 | 0.335 | 0.503 | 0.046 | 0.344 | 0.176 | 0.197 | 0.209 | 0.030 |
| BL1-30 | Lsou34 | NA | 0.619 | 1.000 | 0.064 | 1.000 | 0.374 | NA | NA | NA | 0.143 |
| BL1-30 | Lsou05 | 0.759 | 0.326 | 0.526 | 0.586 | 0.649 | 0.497 | 0.578 | 0.803 | 0.724 | 0.698 |
| BL1-30 | LleA-0 | 0.938 | 0.969 | 0.213 | 0.439 | 0.806 | 0.359 | 0.511 | 0.516 | 0.852 | 0.756 |
| BL1-30 | CtoF-1 | NA | NA | 0.276 | NA | NA | NA | NA | NA | NA | 0.276 |
| BL1-30 | CtoA-2 | 0.179 | 0.447 | 0.481 | 0.872 | 0.068 | 1.000 | 0.566 | 1.000 | 0.765 | 0.467 |
| BL1-30 | BL1-T2 | 0.810 | 0.920 | 0.545 | 0.323 | 0.743 | 0.285 | 1.000 | 0.951 | 0.972 | 0.966 |
| BL1-30 | BL1-2b | NA | 0.762 | NA | 1.000 | NA | 1.000 | 1.000 | NA | 1.000 | 0.948 |
| BL1-61 | LleC-0 | 0.823 | 0.641 | 0.401 | 0.852 | 0.197 | 1.000 | 0.965 | 0.823 | 0.256 | 0.786 |
| BL1-61 | LceC1 | 1.000 | 1.000 | 0.980 | 0.729 | 0.078 | 1.000 | 0.627 | 0.427 | 0.498 | 0.768 |
| BL1-61 | Ca1 | 0.570 | 1.000 | 0.238 | 0.465 | 0.206 | 0.717 | 0.827 | 0.146 | 0.036 | 0.063 |
| BL1-61 | Rser10 | 0.823 | 1.000 | 0.121 | 0.145 | 0.812 | 1.000 | 0.877 | 0.566 | 0.034 | 0.222 |
| BL1-61 | N7K4 | 0.136 | 0.570 | 0.625 | 0.841 | 0.020 | 0.648 | 0.666 | 0.645 | 0.585 | 0.364 |
| BL1-61 | Rru4 | 0.217 | 1.000 | 0.476 | 0.567 | 0.702 | 0.330 | 0.901 | 0.081 | 0.085 | 0.185 |
| BL1-61 | Lsou34 | NA | 1.000 | 0.251 | 0.424 | 1.000 | 0.369 | NA | NA | NA | 0.325 |
| BL1-61 | Lsou05 | 0.861 | 0.525 | 0.995 | 0.036 | 0.621 | 0.717 | 0.025 | 0.482 | 0.760 | 0.256 |
| BL1-61 | LleA-0 | 0.570 | 0.858 | 0.290 | 0.912 | 0.957 | 0.358 | 0.324 | 0.800 | 0.270 | 0.598 |
| BL1-61 | CtoF-1 | NA | NA | 1.000 | NA | NA | NA | NA | NA | NA | 1.000 |
| BL1-61 | CtoA-2 | 0.400 | 1.000 | 0.418 | 0.211 | 0.368 | 1.000 | 0.767 | 0.341 | 0.603 | 0.397 |
| BL1-61 | BL1-T2 | 0.336 | 0.856 | 0.684 | 0.576 | 1.000 | 0.037 | 0.762 | 0.221 | 0.392 | 0.448 |
| BL1-61 | BL1-2b | NA | 1.000 | NA | 0.517 | NA | 1.000 | 0.194 | NA | 0.169 | 0.264 |
| LleC-0 | LceC1 | 1.000 | 0.954 | 0.261 | 0.566 | 0.196 | 1.000 | 0.825 | 0.641 | 0.307 | 0.615 |
| LleC-0 | Ca1 | 0.398 | 1.000 | 0.461 | 0.935 | 0.973 | 1.000 | 0.675 | 0.466 | 0.976 | 0.983 |
| LleC-0 | Rser10 | 0.133 | 0.527 | 0.594 | 0.783 | 0.839 | 0.568 | 0.381 | 0.082 | 0.090 | 0.125 |
| LleC-0 | N7K4 | 1.000 | 1.000 | 0.201 | 0.018 | 0.465 | 1.000 | 0.238 | 0.022 | 0.599 | 0.058 |
| LleC-0 | Rru4 | 0.414 | 0.834 | 0.431 | 0.962 | 0.004 | 0.677 | 0.513 | 0.567 | 0.391 | 0.338 |
| LleC-0 | Lsou34 | NA | 0.790 | 1.000 | 0.803 | 1.000 | 1.000 | NA | NA | NA | 0.923 |
| LleC-0 | Lsou05 | 0.538 | 0.817 | 0.786 | 0.561 | 0.162 | 0.913 | 0.403 | 1.000 | 0.624 | 0.700 |
| LleC-0 | LleA-0 | 0.389 | 0.612 | 0.401 | 0.215 | 0.784 | 1.000 | 0.527 | 0.227 | 0.651 | 0.422 |
| LleC-0 | CtoF-1 | NA | NA | 1.000 | NA | NA | NA | NA | NA | NA | 1.000 |
| LleC-0 | CtoA-2 | 1.000 | 1.000 | 0.751 | 0.247 | 0.502 | 0.573 | 0.645 | 1.000 | 0.228 | 0.651 |
| LleC-0 | BL1-T2 | 1.000 | 0.479 | 0.915 | 0.483 | 1.000 | 0.272 | 0.872 | 0.817 | 0.756 | 0.966 |
| LleC-0 | BL1-2b | NA | 1.000 | NA | 1.000 | NA | 1.000 | 0.134 | NA | 0.137 | 0.318 |
| LceC1 | Ca1 | 0.709 | 0.438 | 0.420 | 0.002 | 0.649 | 0.144 | 0.529 | 0.847 | 0.776 | 0.184 |
| LceC1 | Rser10 | 0.166 | 0.289 | 0.202 | 0.094 | 0.746 | 0.659 | 0.542 | 0.474 | 0.004 | 0.022 |
| LceC1 | N7K4 | 1.000 | 0.903 | 0.881 | 0.620 | 0.105 | 0.658 | 0.829 | 0.365 | 0.163 | 0.490 |
| LceC1 | Rru4 | 1.000 | 0.661 | 0.903 | 0.919 | 0.523 | 1.000 | 0.073 | 0.945 | 0.516 | 0.906 |
| LceC1 | Lsou34 | NA | 0.854 | 1.000 | 0.808 | 0.450 | 1.000 | NA | NA | NA | 0.846 |
| LceC1 | Lsou05 | 0.573 | 0.721 | 0.232 | 0.978 | 0.428 | 0.258 | 0.708 | 1.000 | 0.110 | 0.584 |
| LceC1 | LleA-0 | 1.000 | 0.905 | 0.693 | 0.119 | 0.533 | 1.000 | 0.945 | 1.000 | 0.356 | 0.867 |
| LceC1 | CtoF-1 | NA | NA | 1.000 | NA | NA | NA | NA | NA | NA | 1.000 |
| LceC1 | CtoA-2 | 0.377 | 1.000 | 0.278 | 0.499 | 0.785 | 1.000 | 0.947 | 0.156 | 0.960 | 0.947 |
| LceC1 | BL1-T2 | 1.000 | 0.113 | 0.061 | 0.188 | 0.539 | 0.314 | 0.609 | 0.927 | 0.440 | 0.172 |
| LceC1 | BL1-2b | NA | 0.404 | NA | 1.000 | NA | 1.000 | 0.484 | NA | 0.400 | 0.446 |
| Ca1 | Rser10 | 0.565 | 1.000 | 0.131 | 0.491 | 0.827 | 1.000 | 0.806 | 1.000 | 0.858 | 0.909 |
| Ca1 | N7K4 | 0.399 | 1.000 | 0.343 | 0.802 | 0.214 | 1.000 | 0.556 | 0.673 | 0.570 | 0.375 |
| Ca1 | Rru4 | 1.000 | 1.000 | 0.527 | 0.784 | 0.781 | 1.000 | 0.355 | 0.087 | 0.746 | 0.703 |
| Ca1 | Lsou34 | NA | 0.281 | 0.769 | 0.793 | 0.683 | 0.625 | NA | NA | NA | 0.533 |
| Ca1 | Lsou05 | 1.000 | 1.000 | 0.030 | 0.938 | 0.785 | 0.070 | 0.391 | 0.677 | 0.524 | 0.378 |
| Ca1 | LleA-0 | 0.548 | 1.000 | 0.582 | 0.607 | 0.677 | 1.000 | 0.377 | 0.789 | 0.784 | 0.751 |
| Ca1 | CtoF-1 | NA | NA | 0.177 | NA | NA | NA | NA | NA | NA | 0.177 |
| Ca1 | CtoA-2 | 1.000 | 1.000 | 0.106 | 0.008 | 0.807 | 1.000 | 0.318 | 0.462 | 0.478 | 0.213 |
| Ca1 | BL1-T2 | 0.922 | 1.000 | 0.077 | 0.900 | 0.088 | 0.504 | 0.223 | 0.504 | 0.144 | 0.051 |
| Ca1 | BL1-2b | NA | 0.498 | NA | 0.279 | NA | 0.619 | 0.533 | NA | 0.959 | 0.744 |
| Rser10 | N7K4 | 1.000 | 1.000 | 0.709 | 0.822 | 0.019 | 1.000 | 0.732 | 0.191 | 0.601 | 0.645 |
| Rser10 | Rru4 | 0.726 | 1.000 | 0.575 | 0.120 | 0.430 | 0.798 | 0.541 | 1.000 | 0.730 | 0.737 |
| Rser10 | Lsou34 | NA | 1.000 | 0.100 | 0.416 | 0.171 | 0.494 | NA | NA | NA | 0.170 |
| Rser10 | Lsou05 | 0.207 | 0.528 | 0.468 | 0.904 | 0.476 | 1.000 | 0.223 | 0.376 | 0.292 | 0.454 |
| Rser10 | LleA-0 | 0.571 | 0.271 | 0.212 | 0.367 | 0.780 | 1.000 | 0.216 | 0.508 | 0.382 | 0.255 |
| Rser10 | CtoF-1 | NA | NA | 1.000 | NA | NA | NA | NA | NA | NA | 1.000 |
| Rser10 | CtoA-2 | 1.000 | 1.000 | 0.798 | 0.830 | 0.288 | 0.771 | 0.640 | 0.785 | 0.023 | 0.624 |
| Rser10 | BL1-T2 | 1.000 | 0.713 | 0.010 | 0.878 | 0.202 | 1.000 | 0.306 | 0.789 | 0.284 | 0.344 |
| Rser10 | BL1-2b | NA | 0.525 | NA | 1.000 | NA | 1.000 | 0.262 | NA | 1.000 | 0.787 |
| N7K4 | Rru4 | 0.420 | 0.608 | 0.770 | 0.945 | 0.472 | 1.000 | 0.265 | 0.246 | 0.629 | 0.705 |
| N7K4 | Lsou34 | NA | 0.270 | 0.633 | 0.605 | 0.033 | 1.000 | NA | NA | NA | 0.241 |
| N7K4 | Lsou05 | 0.532 | 0.616 | 0.866 | 0.928 | 0.582 | 0.449 | 0.839 | 0.714 | 0.770 | 0.904 |
| N7K4 | LleA-0 | 1.000 | 0.312 | 0.578 | 0.930 | 0.929 | 1.000 | 0.678 | 0.829 | 0.319 | 0.809 |
| N7K4 | CtoF-1 | NA | NA | 0.650 | NA | NA | NA | NA | NA | NA | 0.650 |
| N7K4 | CtoA-2 | 0.713 | 0.275 | 0.891 | 0.233 | 0.533 | 0.485 | 0.814 | 0.260 | 0.535 | 0.524 |
| N7K4 | BL1-T2 | 0.808 | 0.731 | 0.370 | 0.482 | 0.118 | 0.324 | 0.989 | 0.368 | 0.402 | 0.342 |
| N7K4 | BL1-2b | NA | 0.349 | NA | 0.287 | NA | 1.000 | 0.552 | NA | 0.253 | 0.311 |
| Rru4 | Lsou34 | NA | 1.000 | 1.000 | 0.943 | 1.000 | 1.000 | NA | NA | NA | 0.998 |
| Rru4 | Lsou05 | 0.751 | 0.447 | 0.038 | 0.883 | 0.960 | 1.000 | 0.715 | 1.000 | 0.715 | 0.807 |
| Rru4 | LleA-0 | 0.311 | 0.614 | 0.696 | 0.126 | 0.425 | 1.000 | 0.777 | 0.514 | 0.824 | 0.657 |
| Rru4 | CtoF-1 | NA | NA | 0.123 | NA | NA | NA | NA | NA | NA | 0.123 |
| Rru4 | CtoA-2 | 0.287 | 0.167 | 0.401 | 0.315 | 0.102 | 0.797 | 0.607 | 0.200 | 0.151 | 0.058 |
| Rru4 | BL1-T2 | 0.560 | 0.663 | 0.804 | 0.344 | 0.859 | 0.575 | 0.592 | 0.405 | 0.799 | 0.885 |
| Rru4 | BL1-2b | NA | 1.000 | NA | 0.161 | NA | 1.000 | 0.483 | NA | 0.729 | 0.464 |
| Lsou34 | Lsou05 | NA | 0.288 | 0.073 | 0.702 | 1.000 | 0.250 | NA | NA | NA | 0.078 |
| Lsou34 | LleA-0 | NA | 0.586 | 0.417 | 0.799 | 1.000 | 0.749 | NA | NA | NA | 0.657 |
| Lsou34 | CtoF-1 | NA | NA | 1.000 | NA | NA | NA | NA | NA | NA | 1.000 |
| Lsou34 | CtoA-2 | NA | 0.380 | 0.490 | 1.000 | 1.000 | 1.000 | NA | NA | NA | 0.602 |
| Lsou34 | BL1-T2 | NA | 0.394 | 1.000 | 0.646 | 1.000 | 0.629 | NA | NA | NA | 0.684 |
| Lsou34 | BL1-2b | NA | 0.214 | NA | 1.000 | NA | 1.000 | NA | NA | NA | 0.398 |
| Lsou05 | LleA-0 | 0.693 | 0.966 | 0.104 | 0.045 | 0.912 | 1.000 | 0.203 | 0.451 | 0.479 | 0.172 |
| Lsou05 | CtoF-1 | NA | NA | 1.000 | NA | NA | NA | NA | NA | NA | 1.000 |
| Lsou05 | CtoA-2 | 0.096 | 0.450 | 0.154 | 0.800 | 0.817 | 1.000 | 0.030 | 0.553 | 0.597 | 0.173 |
| Lsou05 | BL1-T2 | 1.000 | 0.921 | 0.002 | 0.223 | 0.753 | 0.284 | 0.723 | 0.462 | 0.272 | 0.085 |
| Lsou05 | BL1-2b | NA | 0.165 | NA | 0.563 | NA | 0.496 | 0.493 | NA | 0.810 | 0.508 |
| LleA-0 | CtoF-1 | NA | NA | 1.000 | NA | NA | NA | NA | NA | NA | 1.000 |
| LleA-0 | CtoA-2 | 0.850 | 0.456 | 0.037 | 0.614 | 0.315 | 1.000 | 0.114 | 1.000 | 0.338 | 0.092 |
| LleA-0 | BL1-T2 | 1.000 | 1.000 | 0.918 | 0.177 | 0.662 | 1.000 | 0.974 | 0.113 | 0.245 | 0.842 |
| LleA-0 | BL1-2b | NA | 1.000 | NA | 1.000 | NA | 1.000 | 0.802 | NA | 0.850 | 0.996 |
| CtoF-1 | CtoA-2 | NA | NA | 1.000 | NA | NA | NA | NA | NA | NA | 1.000 |
| CtoF-1 | BL1-T2 | NA | NA | 0.594 | NA | NA | NA | NA | NA | NA | 0.594 |
| CtoF-1 | BL1-2b | NA | NA | NA | NA | NA | NA | NA | NA | NA | NA |
| CtoA-2 | BL1-T2 | 0.663 | 1.000 | 0.679 | 0.862 | 0.927 | 0.886 | 0.120 | 0.928 | 0.539 | 0.923 |
| CtoA-2 | BL1-2b | NA | 1.000 | NA | 0.390 | NA | 0.497 | 1.000 | NA | 0.316 | 0.448 |
| BL1-T2 | BL1-2b | NA | 0.060 | NA | 0.086 | NA | 0.620 | 0.642 | NA | 0.009 | 0.0004 |

NA = comparison for which no contingency table was computable.

**Table S5:** Results for the Wilcoxon’s sign rank test computed by BOTTLENECK for each river and for the TPM and SMM microsatellite mutation models. Significant H_e_ excesses are evidences of recent population decreases. Significant H_e_ deficiencies can be interpreted as recent signals of demographic expansion. ^ns^ means that there is not a significant deviation from mutation/drift equilibrium (P > 0.05); ***** indicates a significant deviation from mutation/drift equilibrium (P ≤ 0.05). No significant H_e_ deviation has been found after the application of false discovery rate procedure (Benjamini and Hochberg, 1995).

|  | TPM |  | SMM |  |
| --- | --- | --- | --- | --- |
| Code | Wilcoxon  excess | Wilcoxon  deficiency | Wilcoxon  excess | Wilcoxon  Deficiency |
| AVE | 0.788 ^ns^ | 0.235 ^ns^ | 0.945 ^ns^ | 0.065 ^ns^ |
| BAR | 0.148 ^ns^ | 0.866 ^ns^ | 0.500 ^ns^ | 0.524 ^ns^ |
| CEL | 0.163 ^ns^ | 0.852 ^ns^ | 0.596 ^ns^ | 0.428 ^ns^ |
| ELL | 0.476 ^ns^ | 0.548 ^ns^ | 0.879 ^ns^ | 0.134 ^ns^ |
| HER | 0.122 ^ns^ | 0.892 ^ns^ | 0.446 ^ns^ | 0.580 ^ns^ |
| LOU | 0.643 ^ns^ | 0.380 ^ns^ | 0.892 ^ns^ | 0.121 ^ns^ |
| SAL | 0.500 ^ns^ | 0.527 ^ns^ | 0.706 ^ns^ | 0.318 ^ns^ |
| SAV | 0.259 ^ns^ | 0.765 ^ns^ | 0.604 ^ns^ | 0.425 ^ns^ |
| VIA | **0.047*** | 0.960 ^ns^ | 0.527 ^ns^ | 0.500 ^ns^ |

**Table S6:** Median, 5% and 95% quartile values calculated for N_0_ (the current effective population size), N_1_ (the past effective population size), T_a_ (the time of the beginning of the demographic change, in years backwards from the present) and Log_10_(N_0_/N_1_) (the magnitude of the demographic change) for each river, through the posterior distributions obtained with MSVAR 1.3. Negative values of the ratio Log_10_(N_0_/N_1_) indicates that the population has experienced a bottleneck.

|  | N_0_ |  |  | N_1_ |  |  | T_a_ |  |  | Log_10_(N_0_/ N_1_) | |  |
| --- | --- | --- | --- | --- | --- | --- | --- | --- | --- | --- | --- | --- |
| River | Median | 5%  quartile | 95%  quartile | Median | 5%  quartile | 95%  quartile | Median | 5%  quartile | 95%  quartile | Median | 5%  quartile | 95%  quartile |
| AVE | 18 | 0.26 | 257.93 | 5705 | 1563.72 | 19967.73 | 216 | 5 | 2828 | -1.005 | -2.739 | -0.444 |
| BAR | 22 | 0.35 | 259.72 | 8449 | 2083.62 | 33151.99 | 260 | 6 | 3107 | -0.999 | -2.605 | -0.500 |
| CEL | 20 | 0.38 | 168.39 | 5948 | 1734.11 | 19830.60 | 279 | 8 | 2375 | -1.005 | -2.479 | -0.551 |
| ELL | 58 | 0.42 | 524.68 | 5499 | 1545.67 | 18950.53 | 419 | 5 | 5176 | -0.705 | -2.148 | -0.342 |
| HER | 7 | 0.09 | 77.55 | 9155 | 2506.26 | 32953.91 | 192 | 4 | 1792 | -1.345 | -3.182 | -0.748 |
| LOU | 37 | 0.58 | 513.25 | 5286 | 1506.20 | 18441.77 | 307 | 7 | 4603 | -0.810 | -2.419 | -0.332 |
| SAL | 63 | 1.06 | 401.75 | 5501 | 1426.57 | 20578.35 | 727 | 14 | 6349 | -0.710 | -1.968 | -0.391 |
| SAV | 12 | 0.20 | 119.34 | 6541 | 1630.16 | 26012.09 | 333 | 8 | 3156 | -1.135 | -2.869 | -0.628 |
| VIA | 15 | 0.27 | 142.15 | 6031 | 1652.18 | 20759.73 | 221 | 6 | 2008 | -1.054 | -2.810 | -0.583 |

**Table S7:** Values for the Mann-Kendall's S statistic, variance in S (Var(S)), mean densities and P values obtained for the twelve time series with the modified Mann-Kendall trend test. Time series are identified by the three-letter code of their corresponding rivers. Negative S values denote decreasing trends, while positive values indicate increasing trends. ***** indicates that the trend is significant.

| River | S | Var(S) | Mean density | P |
| --- | --- | --- | --- | --- |
| HER | -25 | 125.0 | 5.9 | **0.025*** |
| VOL | -14 | 65.3 | 13.1 | 0.083 |
| LOU | -18 | 268.7 | 1.8 | 0.272 |
| ARI | 10 | 65.3 | 1.8 | 0.216 |
| VEN | -82 | 697.0 | 10.3 | **<0.01*** |
| GAR | -12 | 125.0 | 0.9 | 0.283 |
| AUR | -37 | 268.7 | 3.8 | **0.023*** |
| ARR | -29 | 268.7 | 1.2 | 0.076 |
| CEL | -29 | 165.0 | 4.0 | **0.023*** |
| VER | -49 | 589.3 | 2.3 | **0.043*** |
| BAR | -2 | 589.3 | 3.8 | 0.93 |
| COU | 23 | 33.8 | 7.4 | **<0.01*** |
